# Exercise Increases Insulin Content and Basal Secretion in Pancreatic Islets in Type 1 Diabetic Mice

**DOI:** 10.1155/2011/481427

**Published:** 2011-09-11

**Authors:** Han-Hung Huang, Kevin Farmer, Jill Windscheffel, Katie Yost, Mary Power, Douglas E. Wright, Lisa Stehno-Bittel

**Affiliations:** ^1^Department of Physical Therapy and Rehabilitation Science, University of Kansas Medical Center, 3901 Rainbow Blvd., Kansas City, KS 66160, USA; ^2^Department of Anatomy and Cell Biology, University of Kansas Medical Center, 3901 Rainbow Blvd., Kansas City, KS 66160, USA

## Abstract

Exercise appears to improve glycemic control for people with type 1 diabetes (T1D). However, the mechanism responsible for this improvement is unknown. We hypothesized that exercise has a direct effect on the insulin-producing islets. Eight-week-old mice were divided into four groups: sedentary diabetic, exercised diabetic, sedentary control, and exercised control. The exercised groups participated in voluntary wheel running for 6 weeks. When compared to the control groups, the islet density, islet diameter, and **β**-cell proportion per islet were significantly lower in both sedentary and exercised diabetic groups and these alterations were not improved with exercise. The total insulin content and insulin secretion were significantly lower in sedentary diabetics compared to controls. Exercise significantly improved insulin content and insulin secretion in islets in basal conditions. Thus, some improvements in exercise-induced glycemic control in T1D mice may be due to enhancement of insulin content and secretion in islets.

## 1. Introduction

Across the globe, the incidence of type 1 diabetes (T1D) is increasing at an alarming rate with the age of onset decreasing within a 20-year period [1]. T1D is an autoimmune disease that leads to impaired glucose homeostasis, because the insulin-producing cells (*β*-cells) located in the pancreatic islets of Langerhans are attacked by the immune system [2]. The clinical benefits attributed to exercise for individuals with type 2 diabetes have been well documented [3–7]. In T1D, far fewer reports have focused on the role of exercise, and yet there are strong indications for decreased risk of diabetes-associated complications including improved arterial function [8–11], cardiac performance [12–17], and lipid metabolism [18] resulting in lower total cholesterol, LDL-c, and triglycerides [19, 20].

Only recently have studies focused on the relationship between exercise and blood glucose regulation in T1D. In general, these reports have shown reduced insulin dosage with increased physical activity [21] along with better glycemic control [22]. In fact, moderate to vigorous activity has been associated with greater overall fitness, a higher fat free mass, and lower glycosylated hemoglobin (HbA1c) levels in people with T1D [19, 20]. In a randomized study of 196 adults with T1D, those that exercised moderately once to three times per week significantly reduced the HbA1c levels and insulin requirements [23]. Even more convincing is a large cross-sectional study of over 19,000 children with T1D, finding that the amount of physical activity was one of the strongest factors predicting lower HbA1c values [24]. 

In spite of these impressive results, little is known about the mechanism of action to explain the lowered blood glucose levels with exercise. There are two general sites where exercise could directly affect blood glucose regulation: (1) insulin secretion of the islets and (2) insulin-stimulated glucose uptake in the skeletal muscle [18, 25, 26]. Only two papers have directly tested the first possibility: changes in insulin levels with exercise in T1D. Unfortunately, the only assay used was an immunohistochemical measurement of the number of hormone-positive cells in the islets of exercised diabetic rats [27, 28]. Clearly, more must be done to begin to unravel the cellular changes occurring in islets with exercise training. We utilized a T1D animal model to examine islet morphology, density, size, cell composition, insulin secretion, and insulin content after 6 weeks of voluntary exercise. By testing the hypothesis in an extremely severe form of T1D without insulin treatment, we ensured that any changes we identified in the islets were due directly to the effects of exercise and not secondarily to a reduction of blood glucose levels.

## 2. Materials and Methods

### 2.1. Animals

The animal protocols were approved by the Institutional Animal Care and Use Committee. Fifty-three 8-week-old male A/J mice were divided into four groups: (1) sedentary (nonexercised) diabetic (*n* = 18), (2) exercised diabetic (*n* = 13), (3) sedentary control (*n* = 11), and exercised control (*n* = 11). Both diabetic groups were injected with streptozotocin, toxic to islet *β*-cells, (freshly dissolved in 10 mM sodium citrate, pH 4.5, with 0.9% NaCl, (Sigma, St. Louis, Mo, USA)) on consecutive days (1st injection of 85 mg/kg body weight and 2nd injection of 65 mg/kg body weight) to induce diabetes. Control animals were injected with 0.4 mL of sodium citrate buffer, pH 4.5. Mice were fasted 3 hours before and 3 hours after each injection. Blood glucose levels were monitored weekly (glucose diagnostic reagents; Sigma) after fasting for 2 hours. Mice in the two diabetic groups were identified as having diabetes when blood glucose levels reached greater than 190 mg/dL.

### 2.2. Exercise

After the induction of diabetes, the mice from the exercise groups were individually housed in cages containing exercise wheels (Mini Mitter Co. Inc., a Respironics Company, Bend, Ore, USA) for 6 weeks starting 3 days after the 2nd injection of streptozotocin. Each wheel revolution was continuously recorded and summarized in thirty-minute intervals with the Vital View Data Acquisition System (Mini Mitter Co. Inc.) throughout the duration of the study. At the termination of the study, mice were overanesthetized with avertin after a 2-hour fast and the pancreata either processed for immune-staining or for isolated islet retrieval.

### 2.3. Pancreatic Sections

The pancreata were rapidly harvested and fixed in 10% normal buffered formalin. The tissues were subsequently dehydrated in graded concentrations of ethanol, cleared in xylene, and subsequently embedded in paraffin wax at 55°C. The tissues were sectioned at 8 *μ*m thickness, mounted on Superfrost/Plus microscope slides (Fisher Scientific, Pittsburg, Pa, USA, no. 12-550-15) and dried at 40°C overnight and stored at 4°C until processing. The paraffin-embedded sections were deparaffinized/rehydrated in xylene followed by ethanol and PBS serial rehydration. Antigen retrieval was completed in 0.01 M citrate buffer, pH 6.2, with 0.002 M EDTA for 30 min using a steamer. Cells were permeabilized in 1.0% Triton X-100 in 0.1 M PBS, pH 7.4 for 30 min. Hematoxylin and eosin (H&E) staining was performed on some sections to illustrate the general islet morphology under light microscopy.

### 2.4. Immunofluorescence Staining

 Sections were blocked in 10% normal donkey serum, 1.0% bovine serum albumin (BSA), and 0.03% Triton X-100 diluted in 0.1 M PBS, pH 7.4 for 30 min. Incubation with the primary antibody mix was performed at 4°C overnight in a wet chamber followed by incubation with the mix of fluorophore-conjugated secondary antibody at room temperature for 2 hr in a wet chamber protected from light. Both primary and secondary antibodies were diluted in 1% NDS, 1% BSA, and 0.03% Triton X-100. Slides were mounted with antifading the agent Gel/Mount (Biomeda, Foster City, Calif, USA, no. M01). The following primary antibodies were used: anti-insulin (1 : 100, Abcam, Cambridge, Mass,USA, no. ab7842), antiglucagon (1 : 200, Abcam, no. ab10988), and antisomatostatin (1 : 200, Abcam, no. ab53165). Corresponding secondary antibodies were conjugated with Cy2 (1 : 200, Jackson ImmunoResearch Laboratories Inc., West Grove, Pa,USA, no. 706-225-148), Alexa 555 (1 : 400, Molecular Probes, Eugene Ore,USA, no. A31570), and Alexa 647 (1 : 400, Molecular Probes, no. A31573). Images were collected using a Nikon C1si confocal microscope.

### 2.5. Islet Density, Size, and Cell Composition

 Islet density was defined as the numbers of islets per microscopic field. Four random fields were selected per section. Twenty-four nonserial sections of pancreatic tissues from 3 animals per group were evaluated. Islet size was estimated by measuring the diameter of the islet under the software Nikon EZ-C1 3.0 FreeViewer. For cell composition analysis, the relative proportion of *α*-,*β*-, or *δ*-cells in each islet was evaluated by counting the number of individual types of cell and dividing by the total sum of endocrine (*α*, *β*, and *δ*) cells per islet. For each group, 88 to 106 islets from nonserial sections of pancreatic tissues from 3 animals were analyzed for both cell size and cell composition. All the evaluations were done by three blinded examiners with a high interrater reliability (intraclass correlation coefficient; ICC > 0.8).

### 2.6. Insulin Content *In Situ*

 To determine the insulin content in the pancreatic islets *in situ*, insulin immunohistochemistry (IHC) was performed on paraffin-embedded pancreatic tissue (Histostain-Plus, Invitrogen) using anti-insulin antibody (1 : 100, Santa Cruz Biotechnology, Inc., no. sc-9168). After staining, slides were dehydrated in xylene and placed on coverslips in Permount mounting medium (Fisher Scientific, no. S15-100). The specificity of insulin immunoreactivity was confirmed by omitting the primary antibodies from some sections. Images were analyzed with Ps Adobe Photoshop CZ4 extended software by determining the average pixel value of staining per islet and per single cell, using our published procedure [29]. Background staining was subtracted from each value. Eighteen to 25 islets were randomly selected from nonserial sections of pancreatic tissues from 3 to 5 animals per group.

### 2.7. Islet Isolation

Islet isolation methods followed our published procedures described in detail [30–32]. Briefly, mice were anesthetized with an intraperitoneal injection of avertin (20 mg/kg). After the peritoneal cavity was exposed, the pancreatic main duct to the intestine was clamped and the pancreas cannulated *in situ* via the common bile duct. The pancreas was distended with collagenase and removed. Islets were gently tumbled, washed, and passed through a sterile 30-mesh stainless steel screen and centrifuged. The pellet was mixed with histopaque, centrifuged, and the islets floating on the gradient were collected and sedimented. Islets were passed through a sterile 40 *μ*m mesh cell strainer with Hank's buffered salt solution (HBSS). After this cleaning process, islets were placed into CMRL 1066 medium containing 2 mM glutamine, 10% fetal bovine serum (FBS), and 1% antibiotic/ antimycotic solution and put into a 37°C culture chamber containing 5% CO_2_.

### 2.8. Insulin Secretion and Content

 Glucose-induced insulin static secretion assays followed our published procedures [31]. Isolated islets were placed in 24-well plate with more than 10 islets per well and assigned to two groups: low glucose (3 mM) and high glucose (16.6 mM). All wells were preincubated for 2.5 hours in RPMI 1640 containing 10% fetal bovine serum and 3 mM glucose in a 37°C containing 5% CO_2_. After preincubation, media was removed from each well and discarded. Low (3 mM as basal condition) or high (16.6 mM) glucose solutions were added, according to the design. After 30 minutes static incubation in the 37°C and 5% CO_2_, the islets were sedimented and the conditioned medium was collected and frozen at −80°C. At the end of the static incubation, the islets were harvested and frozen at −80°C. The total protein in the islets was extracted by sonication in acid ethanol (0.18 M HCl in 95% ethanol). The released insulin and the total intracellular insulin amounts were determined by the ELISA (ALPCO, Salem, NH, USA) as we have published previously [31]. Insulin secretion and content was normalized to islet numbers using previously published standard procedures [33–36] and by volume (islet equivalents) [30–32]. The experiment was replicated 4-5 times per group.

### 2.9. Statistics

 Results were expressed as averages of each group or cell population ± SEM and were compared using ANOVA with Fisher least significant difference, repeated measures ANOVA, or student's *t*-test, depending on the experimental design. Significant differences were defined as *P* < 0.05.

## 3. Results

The exercised diabetic and exercised control mice were given unlimited access to voluntary running wheels for 6 weeks. The average running distance was 2.9 ± 0.3 km/day for the exercised diabetic group and 4.1 ± 0.4 km/day for the exercised control group (*P* < 0.05). In the dependent variables (body weight, fasting glucose levels, islet morphology, insulin content, and insulin secretion), there was no difference in the parameters between the two nondiabetic groups (sedentary control and exercised control). Since the focus of the study was the effects of exercise on islets and no differences were noted between the two control nondiabetic groups, we compared the data from the two diabetic groups (sedentary nonexercised, diabetic mice, and exercised diabetic mice) to the sedentary, nonexercised controls. 

**Figure 1:**
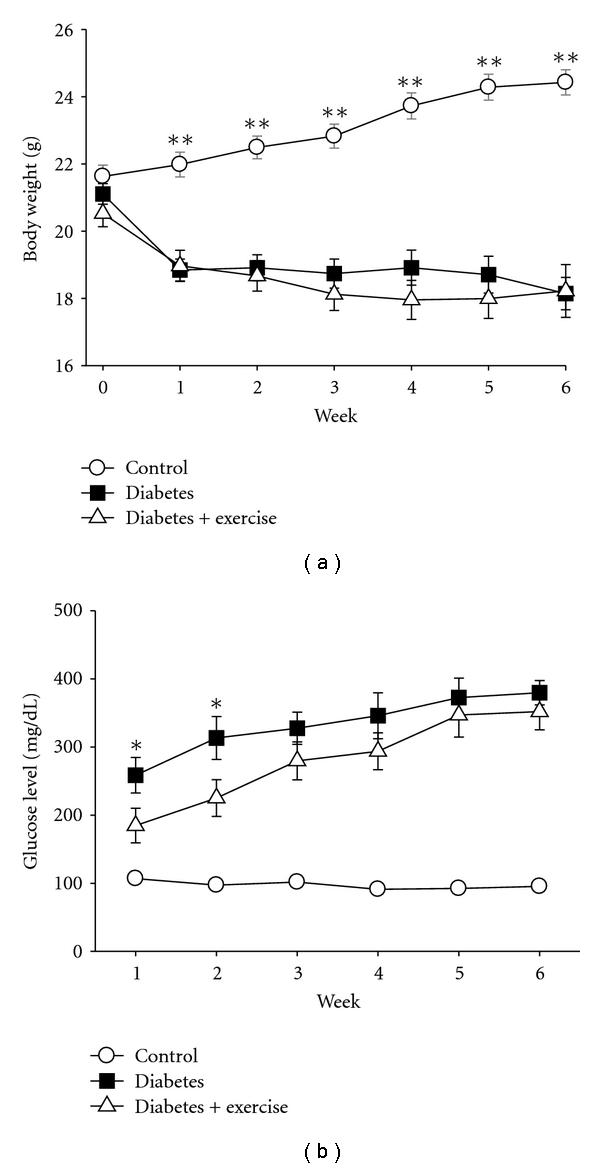
Body weight and glucose levels. (a) Body weight was measured weekly showing a decline in weight with diabetes. **Indicates significant differences (*P* < 0.001) in control (circles) versus the two diabetic groups (squares and triangles). (b) Both diabetic groups had significantly higher blood glucose levels during each week, when compared to the nondiabetic sedentary mice (circles). Blood glucose for the exercised diabetic group (triangles) was lower than the sedentary diabetic group (squares) during the first two weeks. *Indicates significant difference (*P* < 0.05) in nonexercised diabetic versus exercised diabetic groups. Data from all animals in each group were analyzed for the figure at each time point; control (*n* = 11) and sedentary diabetic (*n* = 18) and exercised diabetic (*n* = 13).

Over the 6-week study, the sedentary control group exhibited a 13% increase in body weight. Both diabetic groups exhibited significant reductions in body weight compared to controls (*P* < 0.001). The sedentary and exercised diabetic groups displayed a 14% and 11% reduction in body weight over the 6-week experiment, respectively (Figure 1(a)). 

Fasting glucose levels were significantly higher in both diabetic groups (exercised and sedentary) compared to the controls (*P* < 0.001). Among the diabetic groups, blood glucose levels trended upward over the course of the study to levels above 300 mg/dL. When compared to the sedentary diabetic group, the exercised diabetic group showed significantly lower glucose levels at weeks 1 and 2 (*P* < 0.05) (Figure 1(b)). However, the difference was not statistically significant at later time points.

### 3.1. Islet Morphology

**Figure 2:**
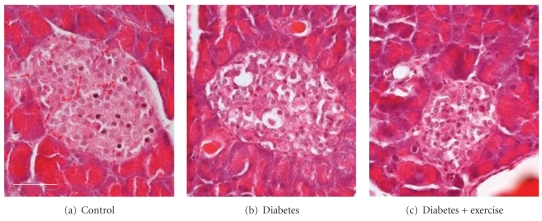
Cellular atrophy and appearance of vacuoles with diabetes. Diabetes was associated with cellular atrophy and increased numbers of vacuoles in the tissue when compared to healthy islets from control animals (a). There were no significant characteristic differences between the sedentary diabetic and the exercised diabetic groups. (Scale bar: 50 *μ*m.)

Cellular atrophy and extensive vacuoles were present in 80% of the islets from sedentary diabetic mice (Figure 2). No overt vacuoles were noted in the tissue samples from the control, nondiabetic animals. The same characteristics (cellular atrophy and vacuoles) were identified in 73% of the islets from the exercised diabetic group. Thus, among diabetic animals, voluntary exercise had no significant effect on the proportion of islets with vacuoles.

### 3.2. Islet Density and Size

**Figure 3:**
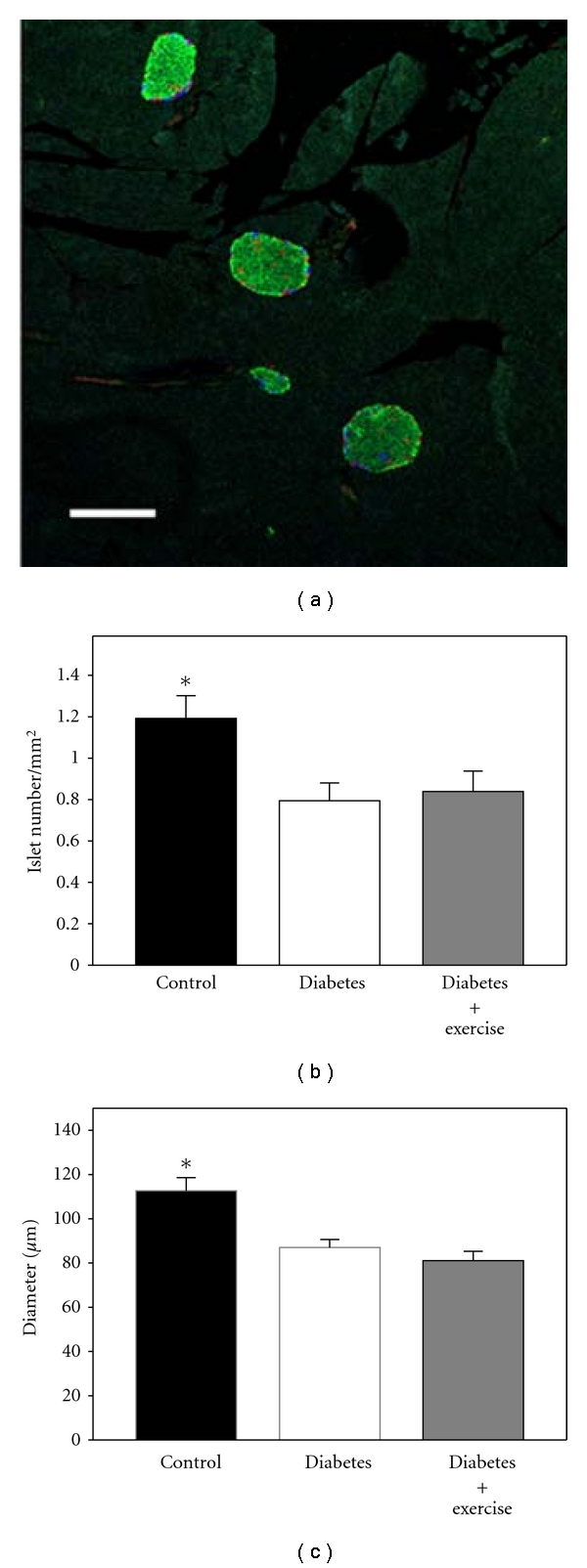
Islet density and size. (a) Four islets are shown in this 10x field of pancreatic tissue from a healthy control animal. (Green: insulin-positive *β*-cells; red: glucagon-positive *α*-cells; blue: somatostatin-positive *δ*-cells.) (Scale bar: 200 *μ*m.) (b) The islet density was determined as the islet numbers normalized per the area of pancreatic section. Exercise did not increase the number of islets per area in the diabetic groups. (c) Exercise did not increase the islet's diameter in the diabetic groups. *Indicates significant differences (*P* < 0.05) between control and the two diabetic groups.

Diabetes negatively affected the number of islets per area, as the islet density was significantly lower in the two diabetic groups compared to the control (*P* < 0.05). Typical islet density from a control animal is shown in the pancreatic section triple stained for insulin, glucagon, and somatostatin (Figure 3(a)).  Figure 3(b) summarizes the islet density for sedentary control, diabetic, and exercise-trained diabetic animals. The sedentary diabetic and exercised diabetic mice had significantly lower islet densities than controls (33% and 30%, resp.). There was no statistically significant difference between two diabetic groups.

Diabetes caused a significant decrease in the diameter of the individual islets in both the exercised and sedentary groups (Figure 3(c)). The sedentary diabetic group had a 23% smaller islet size (diameter) compared to control animals, while the exercised diabetic group had 28% smaller islets compared to the nondiabetic controls (*P* < 0.05).

### 3.3. Islet Cell Composition

**Figure 4:**
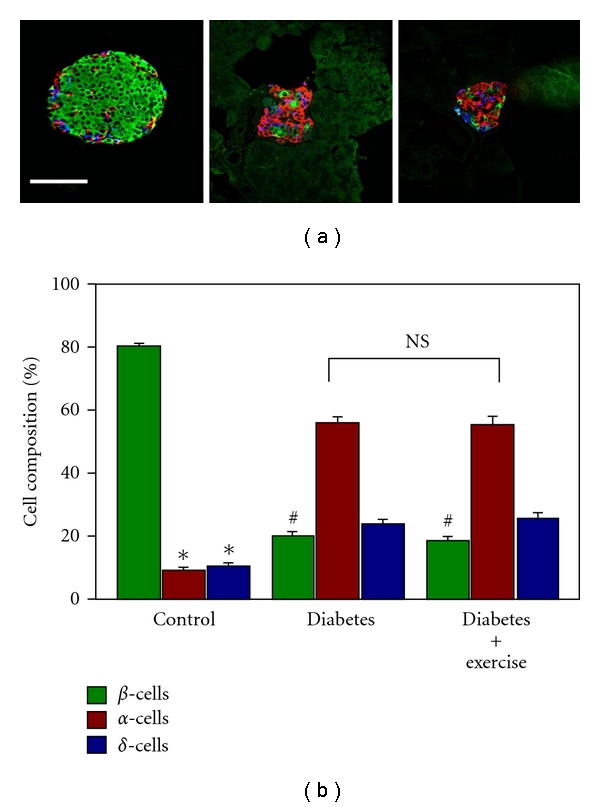
Islet cell composition. (a) Representative islets from each group are shown. In the healthy control (left image), insulin-positive *β*-cells (green) are the most common cell type. However, in both diabetic groups, [diabetic (center image) and diabetic + exercise (left image)] glucagon-positive alpha cells were the major cell type. (Scale bar: 100 *μ*m.) (b) Graph showing the relative proportion of three major types of endocrine cells in the islets from each group. There was no significant difference in the cell composition between the sedentary diabetic and exercised diabetic groups (NS). *Indicates significant difference (*P* < 0.05) in *β*-cells (green) versus *α*-cells (red) and *δ*-cells (blue) in the control. ^#^Indicates significant difference (*P* < 0.05) in *β*-cells (green) between control and diabetic groups.

Pancreatic islets in mammals are composed of several types of endocrine cells, primarily insulin-producing *β*-cells, glucagon-producing *α*-cells, and somatostatin-producing *δ*-cells. Immunofluorescent staining was used to determine the cell composition of individual islets (Figure 4(a)). In islets from the control group, *β*-cells comprised the major (80%) cell type. However, in the islets from the diabetic animals, the alpha cells were the majority (56%) cell type (Figure 4(a)). The percentage of insulin-positive, glucagon-positive, and somatostatin-positive cells from each group of animals is shown in Figure 4(b). The percentage of insulin-positive cells was significantly (*P* < 0.05) lower in islets from sedentary diabetic rats (20.0 ± 1.4%) as compared to the controls (80.4 ± 1.0%). The percentage of glucagon-positive cells was significantly higher (*P* < 0.05) in islets from sedentary diabetic rats (56.0 ± 2.0%) as compared to the controls (9.2 ± 0.7%). Finally, the percentage of somatostatin-positive cells was also significantly higher (*P* < 0.05) in islets from sedentary diabetic rats (24.0 ± 1.2%) as compared to the controls (10.4 ± 0.8%). The cell composition was nearly identical between the sedentary and exercise-trained diabetic animals (NS, Figure 4(b)).

### 3.4. Insulin Content

**Figure 5:**
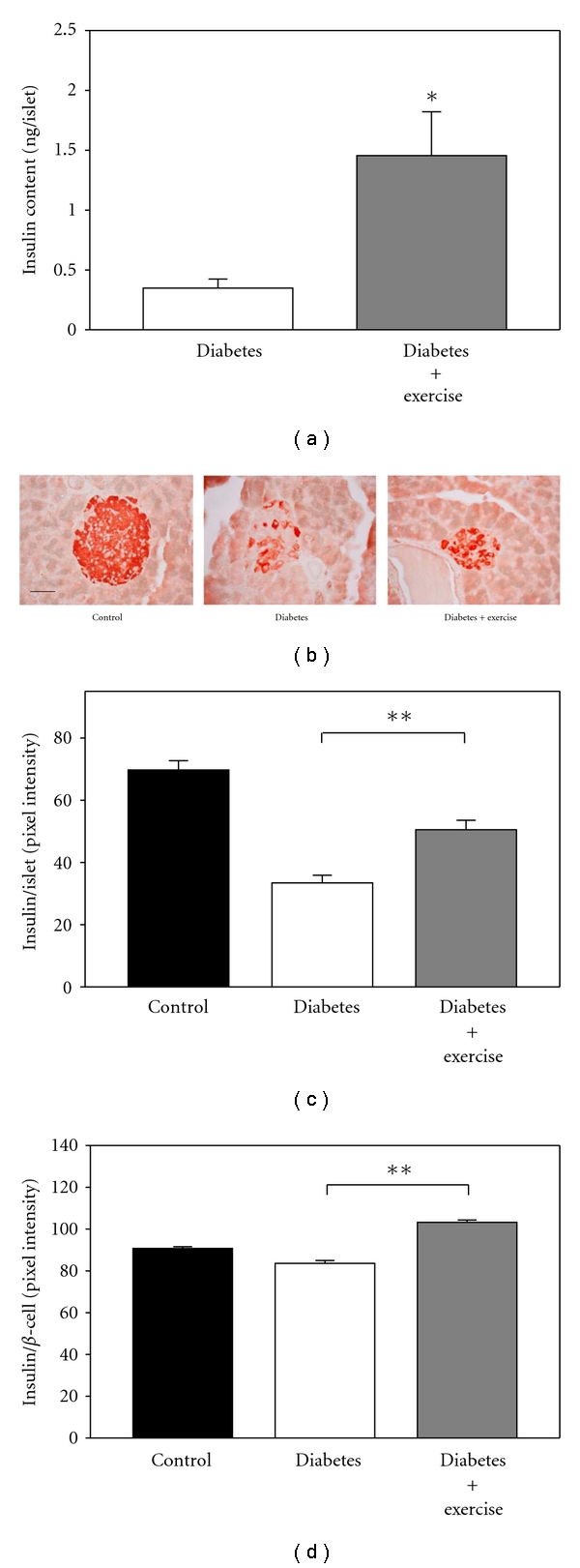
Insulin content per islet. (a) Exercise significantly improved the insulin content per islet between the two diabetic groups (*P* < 0.05). (b) Immunohistochemical staining showing the intensity of insulin-immunoreactive (red) cells in islets from pancreatic sections. (Scale bar: 50 *μ*m.) (c) Quantification of the intensity of insulin-positive staining per islet in pancreatic sections. Exercise improved the total insulin staining intensity per islet significantly (**P < 0.001). (d) The intensity of insulin-positive staining was calculated per *β*-cell, and exercise significantly improved the insulin content per cell (**P < 0.001). Control *N* = 76; sedentary diabetic *N* = 43; exercised diabetic *N* = 56.

 Diabetes significantly reduced the total insulin content of isolated islets from the three groups of animals. Insulin content was normalized to islet using previously published procedures [34, 36]. The average insulin content in the control group was 62.0 ± 4.9 ng/islet. For the sedentary diabetic rats, the insulin content was 0.4 ± 0.1 ng/islet. Exercise increased the insulin content by more than 3 times over the sedentary diabetic animals (1.5 ± 0.4 ng/islet).  Figure 5(a) compares only the two diabetic groups as the control value was too large to effectively fit on the scale. 

In addition to the *in vitro *measurements, we analyzed insulin content *in situ* using published procedures [28, 29]. First, insulin immune reactivity per islet was calculated using published techniques [37]. In the islets from control animals, there was strong insulin staining in all *β*-cells (Figure 5(b)). However, in the sedentary diabetic groups, there was a weak insulin immunoreactivity and it was found in only a few *β*-cells within the islets. Interestingly, in the exercised diabetic group, there was an increase in the insulin labeling intensity (Figure 5(c)). As shown in Figure 5(c), the intensity of insulin-positive staining per islet in the exercised diabetic group was significantly higher compared to the sedentary diabetic group (*P* < 0.001). Exercise improved the total insulin content per islet from 48% to 72% when normalized to the control levels. Further analysis of the insulin intensity per *β*-cell also showed significantly higher insulin intensity in the exercised diabetic group compared to the sedentary diabetic group. Exercise improved the individual cell insulin label intensity from 92% to 114% when normalized to the controls (Figure 5(d); *P* < 0.001).

### 3.5. Insulin Secretion

**Figure 6:**
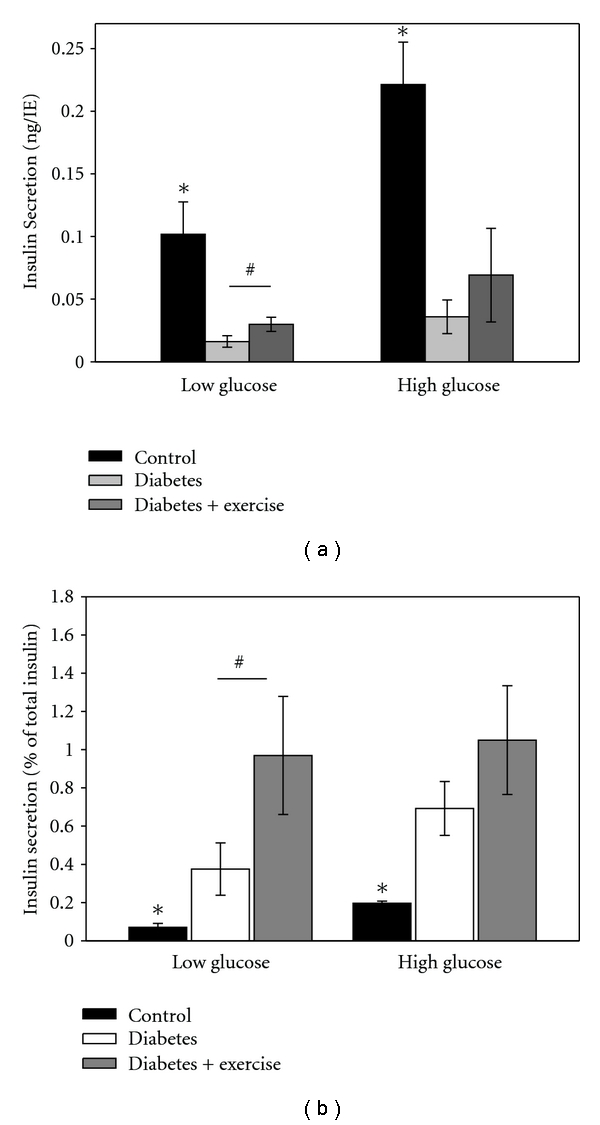
Insulin secretion per islet. (a) Insulin secreted from isolated islets in low (3 mM) or high (16.6 mM) glucose was collected for 30 minutes. Under low glucose conditions, exercise improved the insulin secretion per islet significantly (^#^ *P* < 0.05). Under high glucose, there was no effect of exercise. *P < 0.05 control versus both diabetic groups. (b) When the insulin release was normalized for the total insulin content, exercise increased the percent of insulin released in basal conditions (^#^ *P* < 0.02). With high glucose exposure, exercise induced a change in the insulin secretion, although it did not reach statistical significance.

The glucose stimulated insulin secretion was measured in low (3 mM) or high (16.6 mM) glucose conditions for 30 minutes. Under low glucose, the insulin secretion in the two diabetic groups was significantly lower than the control group (Figure 6(a), *P* < 0.001). Interestingly, the insulin secretion in the exercised diabetic group was significantly (*P* < 0.05) higher when compared to the sedentary diabetic group (Figure 6(a)). Under high glucose, the insulin secretion in the diabetic groups was again significantly lower than in the control (*P* < 0.001). However, there was no significant difference between the exercised diabetic group and sedentary diabetic group. These results were the same whether insulin secretion was normalized by islet or by volume (islet equivalent). 

When the insulin release was normalized for the total insulin content, exercise increased the percent of total insulin released in basal conditions by 2.5 times (Figure 6(b)). The percent of released insulin in the islets from sedentary diabetic mice was 0.38%, and from exercised diabetic mice it was 0.94% (*P* < 0.02). With high glucose exposure, exercise induced a change in the insulin secretion from 0.69 to 0.98% of the total insulin content, although this change did not reach statistical significance. 

## 4. Discussion

In the 1950s, Joslin first suggested that exercise should be an essential component to regulate blood glucose levels of people with T1D, along with a restricted diet and insulin therapy [38]. Yet, today the mechanisms by which exercise regulates blood glucose in conditions of T1D are unclear. In the 1980s, classic work by Reaven and Chang showed that exercised rats with T1D had lower plasma glucose and triglyceride levels than their sedentary counterparts [39]. They hypothesized that the results were due to improved peripheral insulin sensitivity; however, no direct measure of islet mass or insulin content was reported. The current study is the first to measure exercise-induced increases in insulin content and secretion in isolated islets from diabetic animals. 

We hypothesized that exercise could act by protecting islets during the onset of the disease, because in animal models of type 2 diabetes, long-term aerobic exercise resulted in increased islet *β*-cell proliferation, increased *β*-cell mass, and a partial sparing of the abnormal islet morphology noted in the sedentary diabetic rats [40]. Exercise blocked the age-associated morphological changes in the pancreas, including multilobulated, fibrotic islets [41]. In addition, aerobic exercise decreased the presence of proinflammatory cytokines in islet cells [42], but did not change the islet gene expression pattern in type 2 Zucker diabetic fatty rats [43]. 

Morphological examination of the islets from our exercised-trained mice failed to demonstrate differences compared to the sedentary diabetic group. Our findings are similar to a previous report investigating the effect of the exercise on the distribution of *α*-, *β*-, and *δ*-cells and pancreatic polypeptide cells in the islets of streptozotocin-induced diabetic rats [27]. Conversely, another study focused on *β*-cell health and exercise in T1D concluded that exercise partially spared the *β*-cells from diabetes [28]. A difference between the exercise protocols may explain the discordant results. In our protocol and the previous paper by Howarth et al., which showed no change in *β*-cell numbers, the exercise protocol was initiated after the induction of diabetes [27]. In the Coskun et al. study, the aerobic exercise protocol was initiated four weeks prior to the induction of diabetes and the exercise continued for another eight weeks to the termination of the experiment [28]. Thus, exercise may be able to protect *β*-cells if initiated prior to the onset of the disease but has limited or no ability to rescue the *β*-cells once lost.

Even though voluntary exercise did not improve the proportion of insulin-producing *β*-cells in the islets of diabetic animals, it did improve the insulin content in isolated islets. This effect was not an artifact of the isolation procedure, as exercise nearly doubled the total insulin content per islet when examined *in situ*. Our findings are in agreement with a previous publication that showed a beneficial effect on insulin content in diabetic animals after exercise [28]. The previous publication relied solely on insulin immunohistochemistry to draw conclusions. The data presented here confirm that finding, but also demonstrate that exercise was capable of improving the total insulin content and insulin secretion, when measured directly with quantifiable methods. 

One important difference must be highlighted between the previous studies and the current work. In the Coskun et al. paper, the serum glucose measurements were statistically lower in the exercising animals than the sedentary diabetic rats [28]. Thus, one cannot rule out the possibility that any improvements in *β*-cell numbers or function were due to the lowered blood glucose values. In the present study, blood glucose levels were not significantly different between the exercised and sedentary diabetic rats at the termination of the study. This was accomplished by inducing severe diabetes without insulin treatment, and it provided an advantage when interpreting the data, because any changes were directly related to the effects of exercise without a reduction in blood glucose. Since it is known that high glucose is toxic to islet cells [44, 45], maintaining statistically similar blood glucose readings at the termination of the study ensured that glucotoxicity was not a factor. 

Our central finding is that exercise has an effect on the pancreatic islets by stimulating insulin production and/or secretion in diabetic animals. The current results clarify the effect of exercise by showing that the only statistically significant difference in insulin secretion was under low glucose conditions, whether calculated as insulin secretion per islet, per volume (IE), or per total insulin content. While this could be important in maintaining normal blood glucose levels, it would indicate that exercise may not help with postprandial glucose excursions.

## 5. Conclusion

In conclusion, this is the first time that insulin content and secretion has been directly measured in T1D animals following exercise training. In fact, only two other publications have focused on the direct effects of exercise on islets under diabetic conditions [27, 28]. In our study, six weeks of voluntary exercise did not alter islet density, morphology, size or cellular composition. However, insulin content increased *in situ*. Importantly, this is the first study to demonstrate that exercise increased insulin content and improved insulin secretion in isolated islets in uncontrolled T1D.
